# A Male Patient with Hydrocephalus via Multimodality Diagnostic Approaches: A Case Report

**DOI:** 10.34133/cbsystems.0135

**Published:** 2024-07-01

**Authors:** Xiuyun Liu, Jingjing Mu, Meijun Pang, Xuehai Fan, Ziwei Zhou, Fang Guo, Kai Yu, Huijie Yu, Dong Ming

**Affiliations:** ^1^ State Key Laboratory of Advanced Medical Materials and Devices, Medical School, Tianjin University, Tianjin 300072, China.; ^2^ Haihe Laboratory of Brain Computer Interaction and Human-Machine Integration, Tianjin 300380, China.; ^3^ Department of Neurosurgery, Tianjin Medical University General Hospital, Tianjin 300070, China.; ^4^ Department of Neurosurgery, Tianjin Huanhu Hospital, Tianjin 300060, China.

## Abstract

**Introduction:** Idiopathic normal pressure hydrocephalus (iNPH) is a kind of hydrocephalus that is easily to be misdiagnosed with brain atrophy due to the similarity of ventricular dilation and cognitive impairment. In this case, we present an old male patient who was diagnosed with iNPH by multimodality approaches. **Outcomes:** A 68-year-old male patient, with deteriorated gait, declined cognitive function for at least 3 years and urinary incontinence for 3 months. The doctors suspected him a patient with hydrocephalus or Alzheimer's disease based on his symptoms. We used multimodality diagnostic approaches including brain imaging, cerebrospinal fluid tap test, continuous intracranial pressure monitoring, and infusion study to make the final diagnosis of iNPH. He underwent ventriculoperitoneal shunt surgery and was well recovered. **Conclusion:** This case demonstrates the efficacy of using multimodality approaches for iNPH diagnosis, which saves patient time and clinical cost, worthy of further promotion.

## Introduction

Hydrocephalus is caused by abnormal accumulation of cerebrospinal fluid (CSF), and its neurological feature is ventricular dilation. Idiopathic normal pressure hydrocephalus (iNPH) is defined as classic triad of symptoms including cognitive impairment, urinary incontinence, gait disturbance, etc. [1]. The prevalence of iNPH is 1.30% in people over 65 years old and 5.9% in people over 80 years old [2].

However, for some confusing cases, it is difficult to distinguish iNPH with brain atrophy due to the fact that the clinical symptoms of both diseases are very similar [3]. The multimodality diagnostic approaches have great advantages in the diagnosing and treatment of intricate cases. CSF tap test (CSFTT) is considered to be the gold standard for diagnosing hydrocephalus. Recently, more and more imaging techniques appear. In some hospitals in the United Kingdom and France, scientists are doing some researches on the hydrocephalus diagnosis and treatment efficacy of infusion study. Studies have investigated the influence of CSF dynamic perfusion on hydrocephalus or normal pressure hydrocephalus in adults with a ventriculoperitoneal (VP) shunt following subarachnoid hemorrhage [4–6]. Findings suggest that an elevated resistance of CSF (Rcsf) during hydrocephalus points to a disruption in CSF drainage. The infusion study indicates that an Rcsf level exceeding 13 mmHg·min/ml [7] or 18 mmHg·min/ml [8] can be a pivotal physical marker for hydrocephalus diagnosis. In this case report, we combine the multimodality approaches as a whole to make a comprehensive and clear hydrocephalus diagnosis.

A 68-year-old male patient was admitted to Tianjin Medical University General Hospital (Tianjin, China) with deteriorated gait for 7 years, declined cognitive function for 3 years, and urinary incontinence for 3 months. He started to show declined walking ability 7 years ago, characterized by unstable gait, increasing stride frequency and the symptoms of foot drop. The gait problem gradually got worsened in the past 3 years (he fell down more than 30 times because of unsteady walking), accompanied by cognitive impairment, indifferent attitude toward others, decreased communication, and logical analytic abilities (the patient used to be an expert at playing chess, but his playing skill has gradually declined until he completely lost the ability recently). Five years ago, the patient was admitted because of weakness in the left upper limb. He was diagnosed as cerebral infarction because of left central facial paralysis and superficial sensory impairment on the left face. Antiplatelet and statin lipid-lowering therapy was applied to stabilize plaques. Seven months ago, the patient came to the neurology outpatient department for an unknown reason. Magnetic resonance imaging (MRI) showed brain atrophy, and the patient did not receive treatment. Two months ago, he was admitted to the neurology department due to “unstable walking for 7 years and worsening for 1 year”. The diagnosis was (a) Parkinson’s superimposed syndrome, (b) hypertension (grade 2), and (c) ischemic cerebrovascular disease. At discharge, the patient’s condition was stable and was given amlodipine besylate tablets. The symptom of urinary incontinence started to appear (3 to 4 times a month), which seriously affected his quality of life. Therefore, he was admitted to our hospital for further diagnosis and treatment.

There is no history of infectious disease, diabetes, coronary heart disease, blood transfusion, drug or food allergies, and family history of hereditary diseases. He got hypertension 4 years ago and has maintained good blood pressure control with the help of amlodipine (10 mg/d) by peros.

## Materials and Methods

The study was approved and a written consent form was obtained from the patient.

### Brain scans

Patient underwent cranial images that were obtained by MRI (Siemens Prisma 3T, München, Germany). PET brain scans were acquired on the GE-Discovery 710 PET System (GE Healthcare, Chicago, USA) [9].

### CSFTT

A lumbar puncture using a spinal cord needle was used to remove CSF, with the puncture point located at L3/4 gap, and the patient was placed in a transverse position [10]. Sample of approximately 4 to 5 ml of CSF was collected and sent for routine and biochemical examination. Furthermore, patients underwent gait assessment and minimental-state examination before and 24 h after CSFTT. The gait assessment included 5-m timed up and go, 10-m walk test, and timed 180° and 360° turn tests.

### Infusion study

During the CSFTT, we also conducted infusion study via transducer through lumbar puncture, where we pumped saline at a rate of 1ml/min into the lumbar space [11]. The infusing procedure was stopped when intracranial pressure reached a plateau (less than 40 mmHg). The experiment is based on CSF physical modeling, and the data are monitored and recorded by ICM+ (University of Cambridge Enterprise Ltd., Cambridge, UK) [12]. Opening pressure was measured at the site of puncture. During this process, we detected intracranial pressure, Rcsf, brain elasticity, etc.

### Shunt surgery

Electromagnetic-guided right VP shunt surgery under general anesthesia using a tube to drain excess CSF from the brain to the abdominal cavity is operated.

### Statistics analysis

Statistical analysis was used using SPSS (version 24.0, IBM, NY, USA). For all analysis, *t* test was used for normal distributions and nonparametric test for non-normal distributions, and *P* < 0.05 was considered as statistically significant.

## Results

### Positron emission tomography and MRI results

**Fig. 1.**
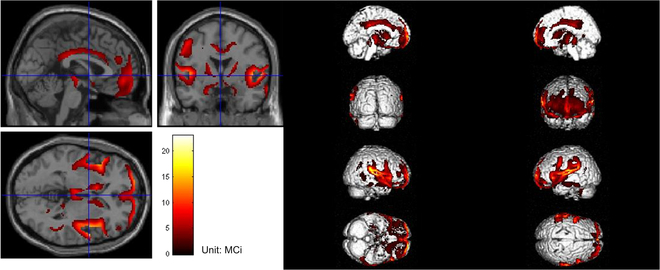
PET scan of a patient with iNPH. The imaging showed that focal decrease in metabolism in the bilateral medial, cingulate gyrus, parietal, insular, and caudate nuclei, bilateral thalamus, and midbrain, especially bilateral ventricles and the peripheral third ventricle.

**Fig. 2.**
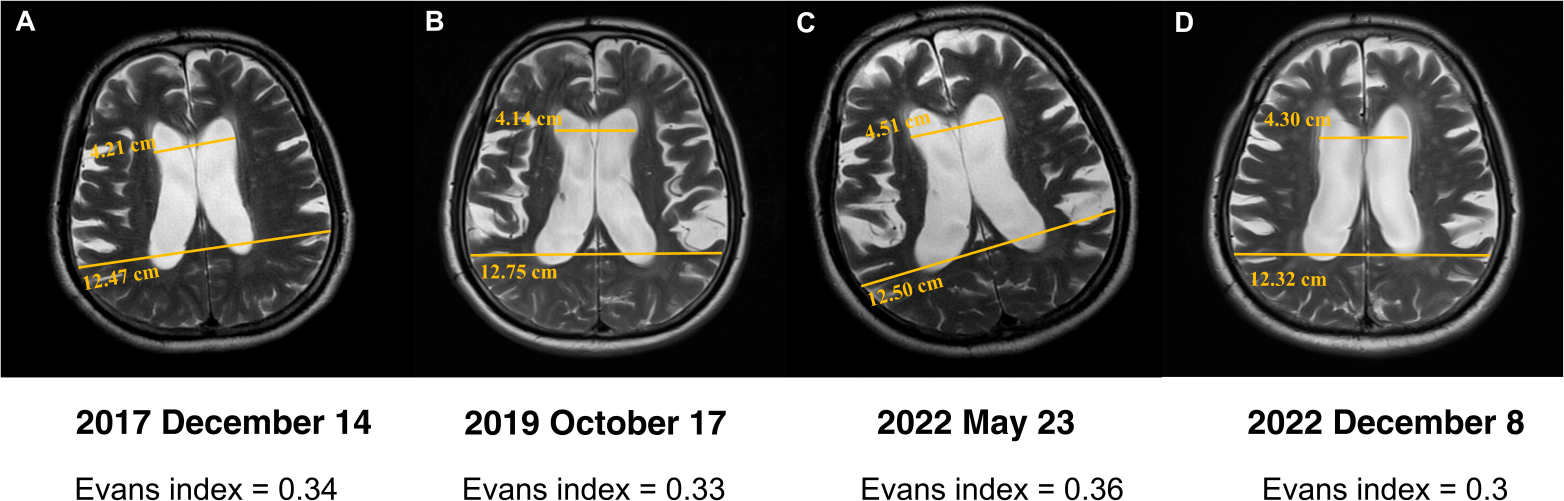
MRI images of a patient with iNPH in chronological order over a 4-year period. Evans index is shown in the picture to demonstrate extent of ventricular enlargement.

The positron emission tomography (PET) scan shows a focal decrease in metabolism in the bilateral medial, cingulate gyrus, parietal, insular, and caudate nuclei, bilateral thalamus, and midbrain, especially bilateral ventricles and the peripheral third ventricle as shown in Fig. 1. His MRI showed progressive enlarged ventricles in the past several years indicated by a corpus callosum angle of <90° and increased Evans index [13] assessed in the year of 2017, 2019, and 2022 (Fig. 2).

### Over 20% increase before and after 24-h CSFTT

The patient underwent lumbar puncture and CSFTT on the second day. The CSF is colorless and transparent, and it showed an increase in red blood cells (10 × 10^6^/l), and Pan’s test was weakly positive. The CSF biochemistry shows that the protein level (0.26 g/l) is within the normal range, and no infection has occurred. As shown in Fig. S1, the data show an over 20% increase in gait balance ability after the CSFTT (Table S1), indicating positive for shunt surgery [14]. In addition, his minimental-state examination score increased from 29 to 30, with minimal changes and good cognitive performance.

### Infusion study demonstrated high Rcsf

As shown in Fig. S2, the opening pressure of the patient was 10.5 mmHg, and the elasticity was 0.22. The patient’s Rcsf was 13.39 mmHg·min/ml, demonstrating blocked circulation of CSF and indicating a positive result for shunt surgery and diagnosis of iNPH.

### Shunt surgery and outcome

The patient underwent electromagnetic-guided right VP shunt surgery. The operation went smoothly, the shunt tube was in good position, and no intraventricular hemorrhage occurred. The patient’s head wound healed successfully, and sutures were removed as planned. The patient was reevaluated for cognitive impairment and urinary evaluation before discharge.

After the operation, the “3 main symptoms of hydrocephalus” were substantially alleviated without unanticipated events, the improvement rate was more than 30%, and the patient was discharged after improvement. MRI imaging of the patient brain 15 months after shunt surgery showed a decreased Evans index, compared with the Evans index before surgery (0.30 versus 0.35), which demonstrates the validity of the diagnosis. The shunt work functions properly until now. The patient signed the informed consent form and was satisfied with this comprehensive diagnosis and treatment.

## Discussion

The misdiagnosis of hydrocephalus and cerebral atrophy has always been an important issue, as both their brain imaging show ventricular dilation. In this case, the patient presented deteriorated gait, which lasted for many years, and he fell down more than 30 times due to unsteady walking. Gradually, he developed cognitive impairment and did not receive treatment for hydrocephalus due to early misdiagnosis of cerebral atrophy, which prolonged the time for correct diagnosis and treatment. Until the onset of urinary incontinence 3 months ago, the patient was admitted to our hospital for further diagnosis.

He was finally diagnosed with iNPH via multimodality diagnostic approaches, including imaging PET, MRI, CSFTT, infusion study, etc. to comprehensively diagnose iNPH. The CSFTT has been used as a routine assessment of hydrocephalus in clinical practice. Compared with traditional CSFTT methods, the infusion study used in this experiment has been a well-defined method to assess the effectiveness or the necessity of proceeding into shunt for iNPH patients. It offers several advantages and alternatives compared to traditional CSFTT, including short-testing duration, calculation of Rcsf, and elasticity [15]. The traditional CSFTT evaluates the patient’s gait and cognition for up to 3 d, and it is subjective as different doctors may draw different conclusions based on their experiences. In this case report, we applied multimodality approaches to diagnosing a sophisticated case, including gait and cognition assessment, MRI imagining, and infusion study, combining the information of the brain structure CSF circulation and clinical symptoms. Specifically, the CSF resistance calculated via infusion study provides an objective description of CSF circulation, which greatly improve diagnostic accuracy. Meanwhile, the multimodality approaches are able to shorten the length of diagnosis from 3 to 1 d, which helps the patients save bedside cost. Therefore, the multimodality diagnosis approaches have great potential to be applied at bedside.

CSFTT has been widely used at bedside for hydrocephalus diagnosis. However, it has several disadvantages including subjective, time-consuming with high rate of misdiagnosis, etc. The result highlights the importance of comprehensive information for precise clinical diagnosis and bedside decision. We wish to provide a step-by-step guideline for clinicians in hydrocephalus diagnosis in the future to help them make more precise decision and clear the clinical doubts. On the basis of a physical model of CSF circulation, infusion study provides an objective and accurate assessment of hydrocephalus patient, which is able to shorten diagnostic time, improve treatment efficiency, and reduce medical expenses, demonstrated by other studies [16,17]. Meanwhile, some suspicious patients with hydrocephalus may not move or do not have consciousness; thus, traditional CSFTT is not suitable for precise diagnosis, in which case, infusion study may be the most appropriate way to diagnose these patients. In the clinical application, for patients who appear enlarged ventricle, gait deterioration, cognitive impairment, or urinary incontinence, the multimodality diagnostic approaches can be used to help doctors diagnose hydrocephalus correctly.

In conclusion, this case demonstrates that iNPH should be comprehensively diagnosed, and our case proved the effectiveness of infusion study as a precise diagnostic tool worth promoting in clinical diagnosis. Multimodality diagnostic approaches provide comprehensive assessments and allow doctors make an objective, fast, and accurate diagnosis of hydrocephalus.

## Ethical Approval

The study was approved by the ethical committee of Tianjin General Hospital (Tianjin, China). Written informed consent was obtained from the patient for publication of this case report and the accompanying images.

## Data Availability

The datasets used and analyzed during the current study are available from the corresponding author (email: xiuyun_liu@tju.edu.cn) on reasonable request.
