# Supplementary material for: A Male Patient with Hydrocephalus via Multimodality Diagnostic Approaches: A Case Report
Source: Cyborg Bionic Syst. 2024 Jul 1;5:0135. doi: 10.34133/cbsystems.0135 (PMC11321655; doi:10.34133/cbsystems.0135)
Supplement: Supplementary 1 — Figs. S1 to S3 Table S1 [file cbsystems.0135.f1.zip › Table.docx]

**Table S1. The result of gait assessment before and after CSFTT.**

|  |  | Before  CSFTT | 24 h After  CSFTT | Improvement  Rate (%) |
| --- | --- | --- | --- | --- |
| 5m TUG | Time (s) | 41 | 16 | 60.98 |
|  | Steps | 34 | 22 | 35.29 |
| 10m walk test | Time (s) | 16 | 11 | 31.25 |
|  | Steps | 28 | 20 | 28.57 |
| Timed 180 °  turn test | Time (s) | 6 | 2 | 66.67 |
|  | Steps | 2 | 2 | - |
| Timed 360 °  turn test | Time (s) | 8 | 4 | 50.00 |
|  | Steps | 8 | 4 | 50.00 |

The stride time after CSFTT showed a higher velocity and more broad-based gait. All parameters demonstrated an increased improvement rate of gait assessment more than 20%. CSFTT, cerebrospinal fluid tap test.
