# Supplementary figures and images for: A Male Patient with Hydrocephalus via Multimodality Diagnostic Approaches: A Case Report
Source: Cyborg Bionic Syst. 2024 Jul 1;5:0135. doi: 10.34133/cbsystems.0135 (PMC11321655; doi:10.34133/cbsystems.0135)

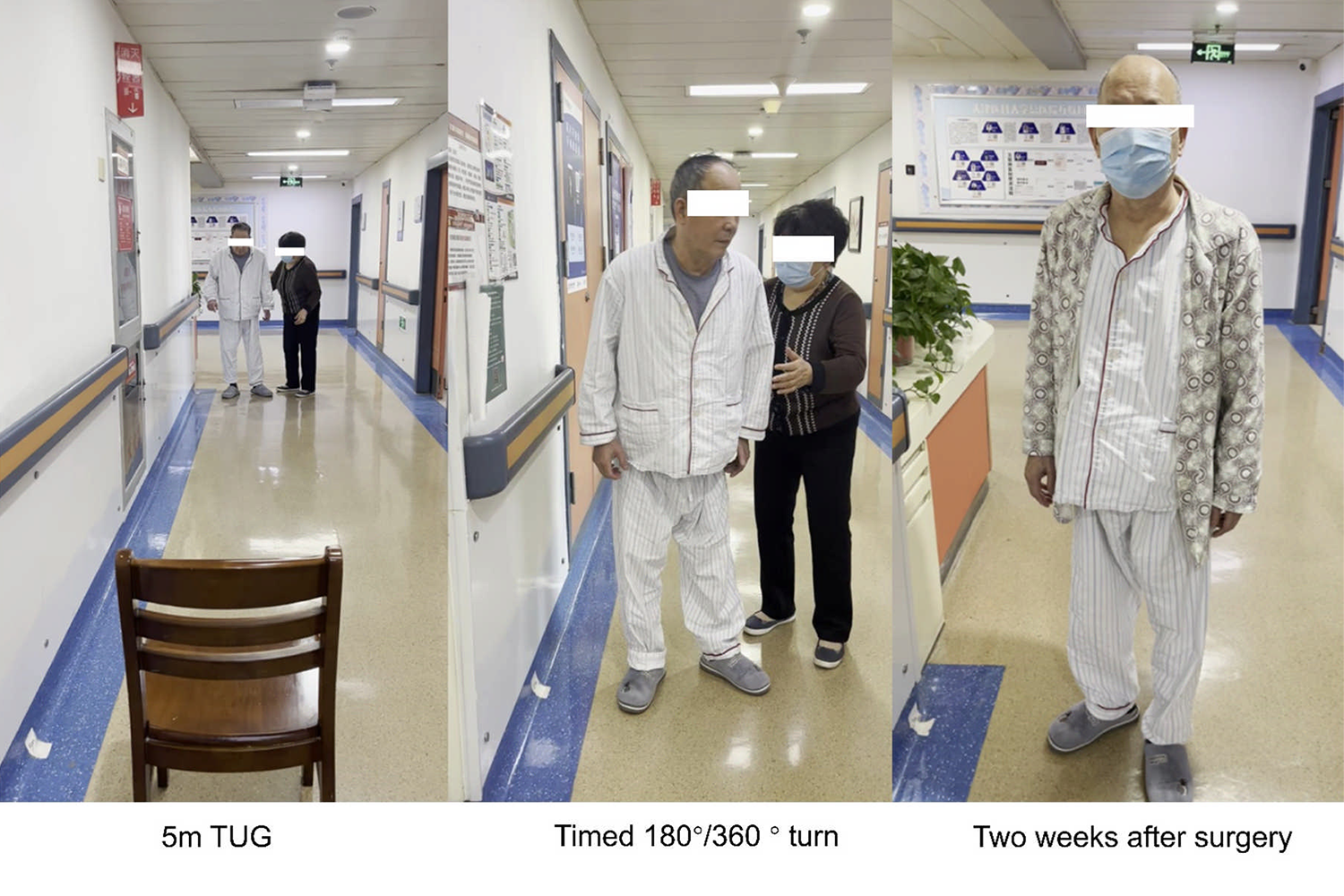

Supplement: Supplementary 1 — Figs. S1 to S3 Table S1 [file cbsystems.0135.f1.zip › Figure S1.tif]

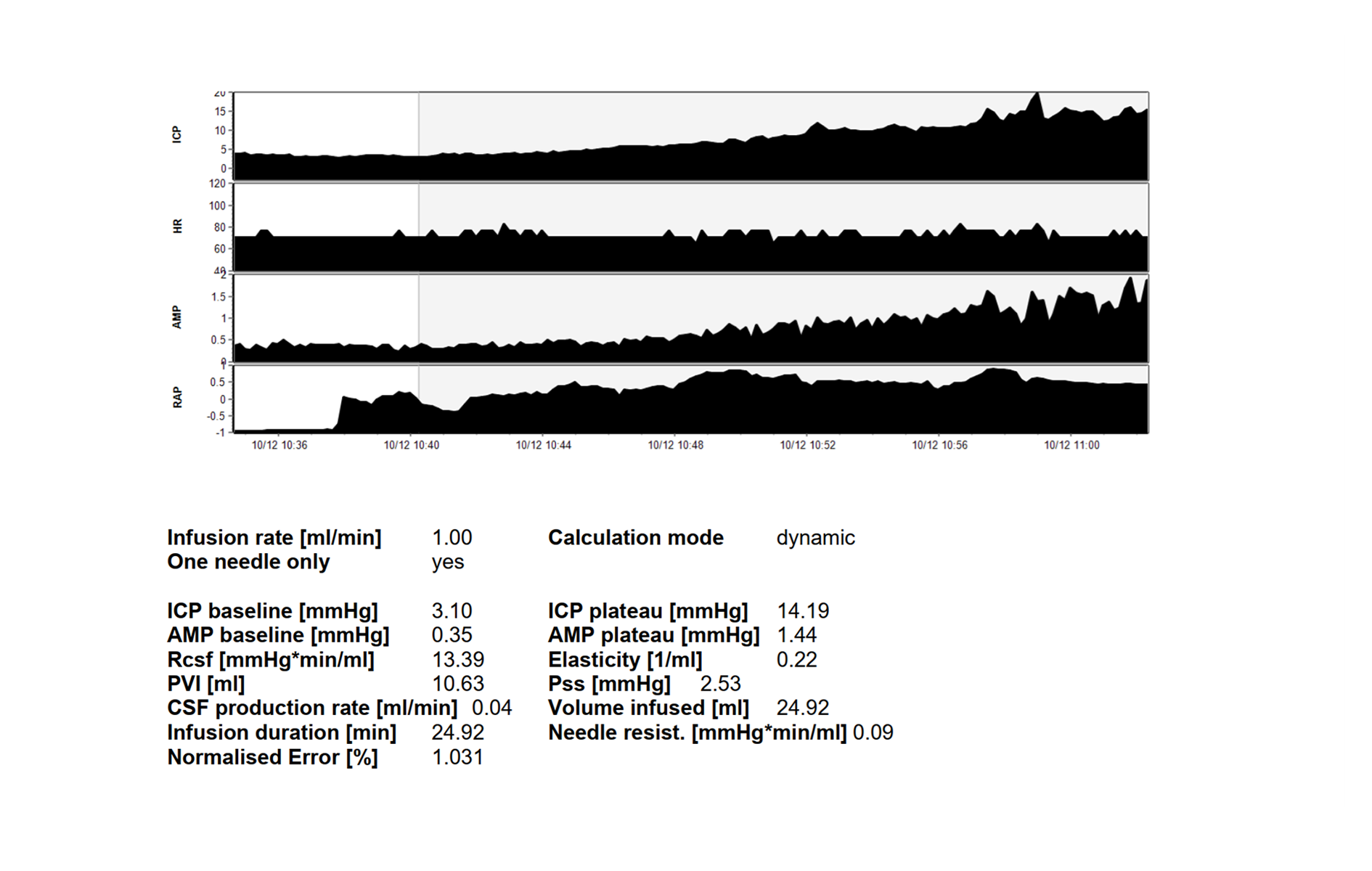

Supplement: Supplementary 1 — Figs. S1 to S3 Table S1 [file cbsystems.0135.f1.zip › Figure S2.tif]

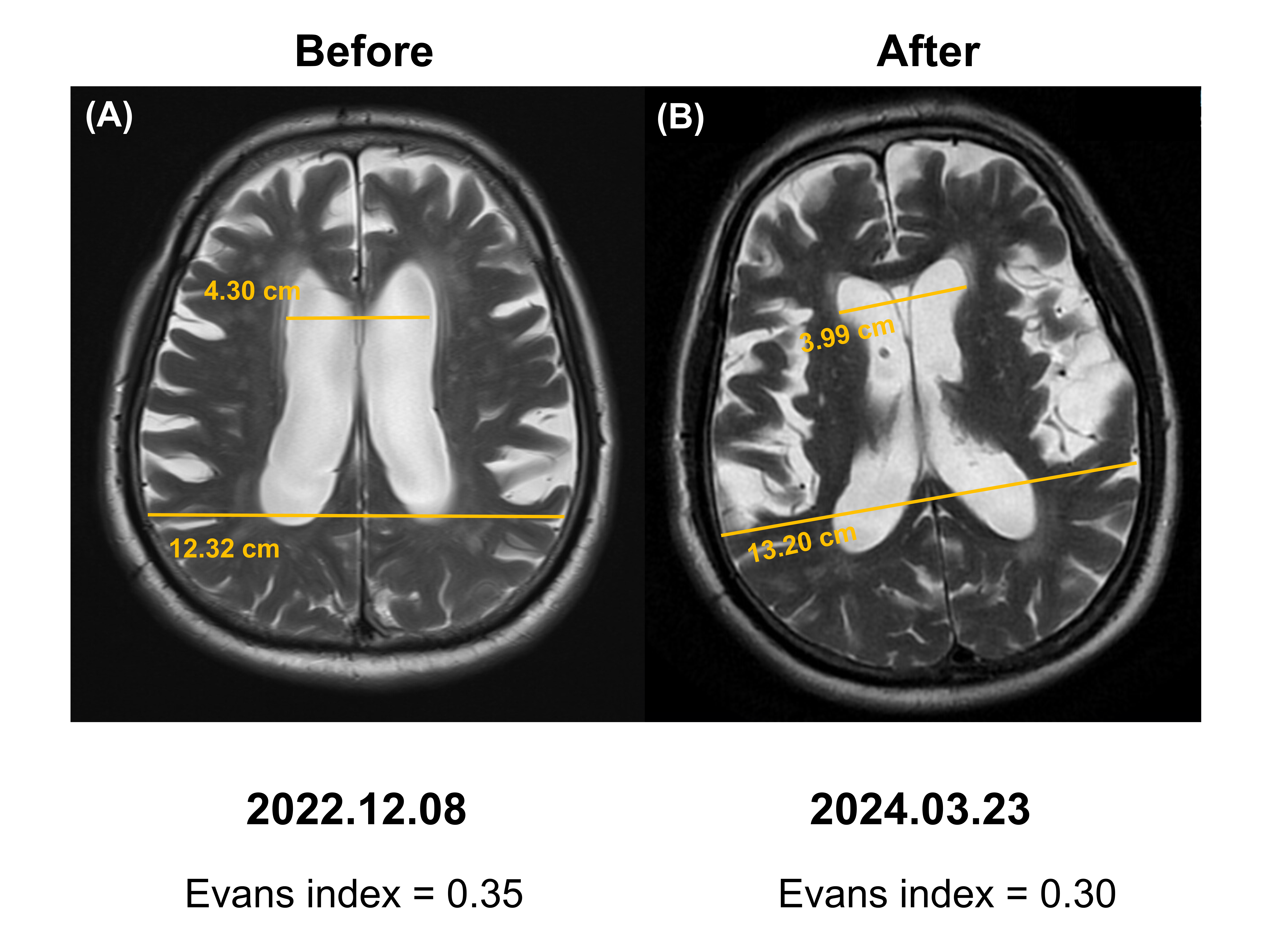

Supplement: Supplementary 1 — Figs. S1 to S3 Table S1 [file cbsystems.0135.f1.zip › Figure S3.tif]
